# The experience of drowning

**DOI:** 10.1177/00258172211053127

**Published:** 2021-11-18

**Authors:** Michael Tipton, Hugh Montgomery

**Affiliations:** 1 Extreme Environments Laboratory, School of Sport, Health & Exercise Sciences, University of Portsmouth, Portsmouth, UK; 2 Centre for Human Health and Performance, University College London, London, UK

**Keywords:** Drowning, cold water, psychophysiology, pathophysiology

## Abstract

Internationally, drowning is a leading cause of accidental death that features in many legal cases. In these cases, possible mitigations and the ‘pain and suffering’ in terms of the duration and subjective experience of drowning are often pivotal in determining levels of compensation and outcome. As a result, there is a requirement to understand the stages of the drowning process, and the duration and physiological and subjective responses associated with each stage. In this short review we focus on these issues.

## Introduction

Drowning is defined as ‘the process of experiencing respiratory impairment from submersion/immersion in liquid’.^1^ It has three outcomes: nonfatal, nonfatal with injury or illness, or fatal: it causes approximately 1000 deaths a day worldwide and leaves many times that number with lifelong morbidity.^2^

The events that result in drowning can be divided into the following sequence: (i) struggle to keep the airway clear of the water, (ii) initial submersion and breath-holding, (iii) aspiration of water, (iv) unconsciousness, (v) cardio-respiratory arrest and (vi) death – inability to revive.

There are many high-quality reviews on the physiology and pathophysiology of drowning.^3–7^ Likewise, drowning following longer term immersion or in diving scenarios, and the treatment of drowning, are dealt with elsewhere.^8,9^ Discussion of these areas does not need to be repeated. Whilst much of this review is applicable to other scenarios, such as warm water, it focuses on the *duration* and *subjective experience* of drowning shortly after immersion in cold water (which we arbitrarily take to be water temperature of less than 15°C). Cold water is a common factor in drowning cases and can be a major determinant of outcome.^10^

From a medico-legal perspective, the questions of ‘how long does it take to drown?’ and ‘what is the pain and suffering associated with drowning?’ are often critical for determining the outcome of drowning-related cases in the courts. This is the primary focus of this short review. However, it is also hoped that this review will also increase the potential to identify a drowning individual, and improve understanding of the impact of time to rescue on outcome.

## Methodology

The literature associated with each of the stages noted above was search, using PubMed and Scopus. It was reviewed using the keywords ‘drowning’, ‘breath holding’, ‘time to unconsciousness’, ‘metabolic rate in water’, ‘subjective experience’, ‘resuscitation’. Only those papers that were directly relevant to the *duration* and/or *subjective experience* of the stages of drowning were considered further. In addition, an internet search of the ‘sensations associated with drowning’ was conducted, and the resulting sites reviewed to determine the relevance and validity of the information provided. It was not possible to completely verify all aspects of the cases presented, but in combination, a common collection of subjective responses emerged. Finally, two medics experienced with treating and debriefing drowning casualties have provided their open-ended input into the experience of drowning.

## The six stages of drowning

### Struggle to keep the airway clear of the water

**Figure 1.**
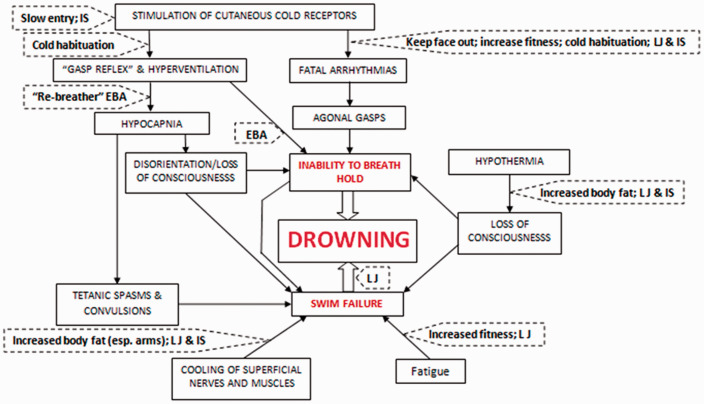
The ‘Physiological Pathways to Drowning’ following immersion/submersion in cold water, with possible interventions for partial mitigation (dashed boxes). IS: immersion suit; LJ: lifejacket; EBA: emergency breathing aid.^8^

In situations where people are forcibly submerged (e.g. sinking craft, ditched inverted helicopter), this phase may be non-existent. It may also be relatively short, 20–60 s, in individuals who cannot swim. In situations where individuals can swim initially, this phase will not start until swim failure begins. This may take hours in warm water and, in this scenario, be associated with exhaustion. In cold water, swim failure can occur, even in good swimmers, in as little as 10 min due to cooling and incapacitation of superficial muscles and nerves^11^ (Figure 1). In this scenario, there is a clear progression towards swim failure in which swim stroke length shortens and stroke frequency increases. The individual becomes more upright in the water and ‘swimming’ becomes ‘treading water’, with periods of submersion and then submersion.^12^

The transition from ‘swimming’ to struggling to keep the airway clear of the water is subtle and often not detected.^13^ Pia^14,15^ studied real-situation film footage to describe the ‘Instinctive Drowning Response’ (IDR) theory (no calling or waving for help, airway repeatedly submerged, upright body, horizontal arm movements just underwater). The IDR is the most widely accepted description of visible drowning behaviour. Pia concluded that drowning people can only struggle on the surface of the water from 20 to 60 s before submersion occurs. This is consistent with the average length of time an individual can work hard anaerobically before exhaustion.^16^ Recently, Carballo-Fazanes et al.,^17^ again using video analysis (23 videos, 52 people, 24 drownings), confirmed some previous observations of drowning behaviour. In this pilot study, the authors identified a large variety of complex visible behaviours of drowning persons, including new behaviours, which mainly included high-frequency resurfacing and backward ‘water milling’. In the small number of videos where sufficient information was available, the interval from the beginning of the incident to the final disappearance ranged between 6 and 95 s (n = 3). The duration of the visible drowning behaviour above the water until it spontaneously stopped, ranged between 76 and 96 s (n = 4). All persons drowned within 2 min.

It is concluded that with swim failure, the struggle to keep the airway clear of the water commences and has characteristic behaviours which should be known to those responsible for rescue. Even if not forcibly submerged, this phase can be very short and is unlikely to last for much longer than 60 to 90 s.

### Initial submersion and breath-holding

The breath-hold time (BHT) following submersion (i.e. when the airway goes beneath the surface of the water) will vary with factors like circumstance (water temperature, clothing worn, exercise undertaken, training, aerobic fitness and experience). There is also substantial inter-individual variability in BHT.

In the laboratory, maximum BHT in warm water, or if well protected with specialist protective clothing in cool water, can approach those seen in air (45 to 60 s). In this scenario, BHT will be primarily determined by a complicated interplay between different influences, including the drive for respiratory movement, the rate of production of carbon dioxide ( $\dot{V}$ CO_2_) and consumption of oxygen ( $\dot{V}$ O_2_).^18^ The amount of exercise undertaken (metabolic rate) determines $\dot{V}$ CO_2_ and $\dot{V}$ O_2_, and thereby influences maximum BHT. End-tidal partial pressures of oxygen (P_et_O_2_) and carbon dioxide (P_et_CO_2_) are normally 100 mmHg and 40 mmHg respectively. At breath-hold breakpoint in air after maximal inhalation, P_et_O_2_ averages 62 mmHg and P_et_CO_2_ 54 mmHg.^19^ Adults cannot normally breath-hold to unconsciousness.

If submerged directly into cold water, BHT is likely to be significantly shorter than that which can be achieved in air. This is due to the respiratory drive evoked by sudden skin cooling and the resulting cold shock response^20^ (Figure 1). For swim-suited, or normally clothed individuals, this response peaks in water somewhere between 10°C and 15°C.^21^ Maximum BHT can be reduced to as little as 0.2 s and average 9.5 s when wearing heavy normal clothing and submerged into 5°C water.^22^ In the same scenario, and even with specialist protective clothing (‘shorty wet suit’ or ‘dry’ suit), maximum BHT can be as short as 1.2 and 8.9 s, respectively,^22^ and average around 20 s in water up to 15°C.^23^ During a simple simulated submerged helicopter underwater escape in water at 10°C, participants wearing a specialist helicopter passenger dry suit and underclothing had an average maximum BHT of 17.2 s.^24^ The corresponding figure for 15°C was 21 s and 20.5 s in water at 5°C.^23^ The insignificant difference in these times between water temperatures is attributed to the high level of immersion-protective clothing worn.

The breakpoint of breath-holding triggers involuntary gasping which, if the airway is submerged, results in the aspiration of water. On immersion in cold water, breath-holding, as noted, is significantly curtailed by a gasp response that can be 2–3L in volume,^21^ that is, greater than the reported lethal volume of aspiration for drowning (see next section). In cold water, the combination of the end of breath-holding and immersion of the face can also result in hazardous cardiac arrhythmias and sudden cardiac death^25^ (Figure 1). This cause of death may be missed at post-mortem as a disturbance to the electrical conductivity of the heart cannot be identified, and agonal gasping may result in the aspiration of water and apparent drowning.

It is concluded that BHT in cold water (5°C) in individuals wearing heavy normal clothing averages around 9.5 s, increasing to an average of around 20 s with a specialist immersion ‘dry’ suit and underclothing.

### Aspiration of water

At the end of breath-holding, and before water is aspirated into the lung, an undetermined percentage of drowning victims (but perhaps up to 89%) swallow water.^4^ Entry of water into the pharynx can cause reflex swallowing, often accentuated by the cough reflex. Transient laryngospasm or bronchospasm may occur as a result of stimulation of the innervated mucosa of the oropharynx and larynx by water. However, it seems unlikely that this situation persists to death (what was previously known as ‘Dry Drowning’^26^). Swallowing of water may explain the high incidence of vomiting in drowning victims.^27^ During and after swallowing, water enters the lung and hypoxia-induced relaxation of the larynx will eventually permit water to be aspirated.

Confusion exists about the volume of water aspirated in drowning.^4^ Aspiration of 2.5 mL . kg^−1^ body weight of sea water causes the pulmonary shunt fraction to increase from about 10% to 75%.^28^ Aspiration of 2.2 mL . kg^−1^ of sea water reduces PaO_2_ from 13 kPa (100 mmHg) to 8 kPa (60 mmHg) within 3 min. The lethal dose of water in the lung has been estimated to be 22 mL . kg^−1^ for salt water, 44 mL . kg^−1^ for fresh water.^29,30^ Salt water may be more ‘lethal’ because more of it remains unabsorbed in the lung, increasing pulmonary pressure and thereby causing earlier right ventricular failure. Often smaller volumes of water (1 to 11 mL . kg^−1^) than those presented above are aspirated in drowning cases.^31–33^ It is worth noting that much of what is known about drowning comes from early work with animals. It is generally acknowledged that, although unpalatable, the data generated are generally applicable to the human condition. Human data are confused by osmotically driven post-mortem changes in the volume of water in the lung and passive aspiration of water.

It is difficult to determine the subjective experience of drowning, and the ‘pain and suffering’ associated with the process, but this is often pivotal in legal cases. The experience of drowning has been reported to be real, profound and transformative.^34^

A review of the reported experiences of those claiming to have drowned was undertaken for this review. The anecdotal testimonies were obtained from different web-based forums and reviewed, assessed for veracity as far as was possible by checking ages of contributors, dates of events, description of events. The testimonies were analysed for content in terms of whether it was probable that water was aspirated into the lung or not, and to identify common experiences between individuals. The initial sift left 34 cases, in 19 of which it was probable that water had entered the lung (aspiration group, AG), and in the other 15 of which aspiration could not be confirmed (non-aspiration group, NAG). In the AG, where reported, the incidents occurred in the sea (n = 3); swimming pools (n = 7); river (n = 1) and lakes (n = 2). The corresponding data for the NAG were: sea (n = 6); swimming pools (n = 3); river (n = 3). Where the precise age at the time of the incident was reported (n = 24), 43% occurred when the individuals were under 10 years of age; 38% when they were 10 to 20 years of age and 19% when over the age of 20 years.

A summary of the subjective comments of those experiencing drowning is presented in Table 1. In both groups, many individuals mention struggling to hold their breath on initial immersion (see previous section). All but two of the AG group (who may have been semi-conscious on submersion) found at least part of the experience ‘painful’, ‘awful’, a ‘struggle’ or ‘shocking’. In all such cases, this was associated with the aspiration of water, irrespective of whether this water was fresh, chlorinated or salt water. A ‘burning sensation’ on aspirating water was specifically mentioned by eight of these individuals. Again, this sensation was not associated with any particular type of water. In contrast, only three individuals in the NAG mentioned their experience being painful; it is possible that these particular individuals aspirated water, but the evidence was not sufficient to place them in the AG. Nine of the NAG (60%) specifically mentioned not feeling any pain.

**Table 1.** Personal accounts of the tranquil perceptions associated with drowning in a range of water temperatures and immersion scenarios.

Respondent	
	Aspiration group
5	I felt at peace.
7	Struggling followed by feeling ‘damn pleasant’.
9	It hurts so badly, then dizzy then calm. Hallucinate.
10	I give in to the lack of oxygen, going limp and at this moment there's no pain or fear. It's just simple and peaceful.
14	I just started breathing. It was quite peaceful not painful. I mean I started thinking, well at least I know that I will die peacefully.
16	I finally inhaled (water). All the tension in my chest instantly cleared and it felt almost as if I were breathing in air. I was cold which was alarming considering the water was warm, but I didn’t exactly care. My whole body had gone lax and I let my eyes slip shut. It felt just like falling asleep.
26	I realized that no one could save me and I began to just relax. I couldn’t move, didn’t want to move. I thought to myself wow this is a stupid way to die. I wonder what happens next. And it’s like the moment I calmed down I couldn’t feel anything. It felt like meditation.
29	Immediately (after inhaling water) you will feel very relaxed and peaceful for a moment or two, also with no fear, until everything goes black and you pass out.
34	It burned at first, but since the water was cold, it soothed me.
	Non-aspiration group
4	My lungs had more or less given out, and there was no pain, just comfort.
5	I felt at peace and knowing that I was gonna die, I wasn’t afraid.
13	Just a second or two later the ‘panic’ feeling left me. The next thing I knew I was looking at a moving wall of beautiful colours; sea shells, sea fish, quiet, beautiful as my body slowly drifted down, down, down. No panic, no pain, no regrets, worries etc., the most pleasant experience I’ve ever had.
19	That peaceful feeling is all part of the euphoria most people feel before death. If you die drowning, I would say it's one of the more peaceful ways to go. After the worst 15 seconds of your life of course.
25	It was more of a numb feeling than anything. Kind of distant, like I was watching everything unfold from a different perspective, like it wasn't really me that was experiencing everything. I think my mind was too dulled by panic and fear and exhaustion to really notice any pain at all.
31	All I can remember was it seems like I was looking through a kaleidoscope of pretty colours. I would’ve disappeared into oblivion peacefully and painlessly.

Five of the AG and three of the NAG reported seeing bright colours under the water during their incident. Five of the AG and three of the NAG felt that time slowed during their incident; this is a common observation during frightening events that may be a function of recollection rather than perception, so not relevant to those who do not survive.^35^ Following a period of struggle to breath hold, and pain on aspirating water, many individuals report much more tranquil perceptions (Table 1).

The sequence of panic and pain followed by more tranquil sensations is supported by those with experience of treating and debriefing drowning victims (e.g. Dr Justin Sempsrott from Lifeguard Without Borders, *personal communication*) who estimate that there is 30–60 s of panic during the surface struggle, then 30–60 s underwater of panic or neutral feelings before loss of consciousness. Others (Dr Frank Golden, *personal communication*) confirm a period of terror while struggling to breath hold, then feeling a tearing, burning sensation in the chest as water enters the airway, followed by, feeling of absolute calmness and tranquillity and a terminal period of stimulation of the CNS then CNS depression and unconsciousness. This sequence is not a new finding, nor is it limited to drowning, being reported in near-death experiences from a wide range of causes.^36^ In 1791, British admiral Sir Francis Beaufort recalled an event in which he drowned: ‘A calm feeling of the most perfect tranquillity succeeded the most tumultuous sensation … Nor was I in any bodily pain. On the contrary, my sensations were now of rather a pleasurable cast’.^36^

Moderate hypoxia (oxyhaemoglobin saturation of the blood 60–80%) does not cause loss of consciousness but does significantly affect the functioning of the brain and the senses. Responses vary from an attitude of serene unconcern, of calm and tranquil indifference to everything, including: pain, hilarity, euphoria or a sense of power with ultimate knowledge.^37^ The calmness, tranquillity and hallucinations arising as part of the drowning process are most likely directly linked to brain hypoxia, alterations in neurotransmitters and consequent cognitive function.^38^ Different parts of the brain have different sensitivities to hypoxia, and the rate at which hypoxia develops varies depending on circumstance (see next section). This, in part, explains the differences in the perceptions experienced by those drowning.

It is concluded that, in addition to the physical effort to keep the airway above the water, followed by the struggle to breath-hold, there is a period of pain, often described as a ‘burning sensation’ as water enters the lung. This sensation appears independent of the type of water (sea, pool, fresh). With time, the sensations of pain and panic can give way to hallucinations and sense of tranquillity, probably associated with the onset of profound hypoxia and impending unconsciousness. These responses and perceptions vary significantly between individuals but appear to represent a common response to the near-death experience of drowning. The timing associated with these events is considered in the following stage.

### Time to unconsciousness

With time, the impairment of gas exchange, and the dilution of surfactant caused by the presence of water in the terminal airways, lead to worsening hypoxia, hypoxemia and, eventually, anoxia. There is depletion of brain energy reserves, failure in brain energy metabolism, deterioration of brain function, loss of consciousness and irreversible neuronal cell injury.^7^

Unconsciousness is generally thought to occur when the oxygen saturation of the blood falls from near 100% to 50 to 60% (P50 on the oxygen dissociation curve i.e. 50% saturation of haemoglobin) or an arterial partial pressure of oxygen (PaO_2_) below 3.6 kPa (27 mmHg) and/or arterial partial pressure of carbon dioxide (PaCO_2_) of 12 to 16 kPa (90–120 mmHg). The critical acute alveolar partial pressure of oxygen (PAO_2_) for hypoxic loss of consciousness in healthy people ventilating normally is between 4.00 kPa (30 mmHg) and 5.07 kPa (38 mmHg).^39^

The time spent at a given PAO_2_ is critical, and this is recognised by including a time factor in the calculation of the ‘dose’ of hypoxia received. Thus, a dose of 20 kPa.s^−1^ (150 mmHg.s^−1^) of hypoxia is thought to be required to induce loss of consciousness,^40–42^ and an acute PAO_2_ of 4 kPa (30 mmHg) would therefore be predicted to result in loss of consciousness in about 5 s.

For a drowning individual, the time taken to become unconscious will depend on the *available oxygen stores of the body* and the *rate of oxygen consumption* (* $\dot{V}$ O_2_*). The $\dot{V}$ O_2_ will, in turn, be determined by factors such as water temperature (increasing $\dot{V}$ O_2_ with falling water temperature), clothing protection worn (decreasing $\dot{V}$ O_2_ with increasing clothing protection) and exercise undertaken (increasing $\dot{V}$ O_2_ with increasing exercise).

The reservoirs of oxygen within the body that are available for metabolism are: dissolved in body fluids; bound to haemoglobin and myoglobin, and in the gas cavities, particularly the lungs. The amount of oxygen available in these stores is usually calculated for a 70 kg human at a lung capacity of 5.5 L in normal atmospheric air (PAO_2_ of 120 mmHg), and ranges between 1546 mL and 2000 mL with an average of 1724 mL. In the drowning scenario, this is likely to represent a maximum value as, on average, 40% of the available oxygen is in the air within the lung, and some of this will be replaced by water with drowning.^43–47^

The $\dot{V}$ O_2_ on immersion in cold water increases above that seen at rest due to increased muscle tension and cardio-respiratory activity caused by sudden cooling of the skin, a part of the cold shock response.^20^ After increasing on initial immersion, $\dot{V}$ O_2_ can fall a little before increasing again due to shivering. The $\dot{V}$ O_2_ is increased further on immersion if physical effort is undertaken, such as struggling to stay afloat or attempting to escape from a ditched vehicle. Published values for $\dot{V}$ O_2_ on immersion in cold water are presented in Table 2. Due to the dynamic nature and attenuation of the $\dot{V}$ O_2_ response during the initial minutes of cold water immersion, data averaged over more than 1 min are likely to underestimate the peak $\dot{V}$ O_2_.

**Table 2.** Oxygen consumption on immersion and submersion in cold water.

Condition	Water temperature (°C)	Clothing	Activity/time	$\dot{V}$ O_2_ (mean + range where provided)(L.min^–1^)	Reference
1	10	Swimming costume	Rest, head-out/first min of immersion	0.99 (range: 0.808 to 1.279)	^48^
2	10	Swimming costume + torso protection	Rest, head-out/first min of immersion	0.916 (range: 0.697 to 1.159)	^48^
3	10	Swimming costume + limb protection	Rest, head-out/first min of immersion	0.882 (range: 0.676 to 1.179)	^48^
4	5	Heavy underclothing + cotton overalls	Simple simulated HUE/first 2 min of immersion	0.972 (range: 0.728 to 1.42)	^22^
5	5	Heavy underclothing + cotton overalls + ‘shorty’ wet suit	Simple simulated HUE/first 2 min of immersion	0.936 (range: 0.749 to 1.218)	^22^
6	5	Heavy underclothing + cotton overalls + immersion dry suit	Simple simulated HUE/first 2 min of immersion	0.77 (range: 0.583 to 0.935)	^22^
7	5	Heavy underclothing + immersion dry suit (Royal Navy winter sea helicopter aircrew assembly)	Simple simulated HUE/first minute of immersion	0.53 (range 0.33 to 0.74)	^23^
8	15	Heavy underclothing +immersion dry suit (Royal Navy winter sea helicopter aircrew assembly)	Simple simulated HUE/first minute of immersion	0.52 (0.4 to 0.59)	^23^
9	10	Swimming costume	Rest, head-out/first 2 min of immersion	0.676	^49^
10	10	‘Normal’ clothing (underwear, socks, trousers, shirt, shoes)	Rest, head-out/first 2 min of immersion	0.586	^49^
11	10	Normal clothing + windproof/waterproof foul weather jacket and trousers.	Rest, head-out/first 2 min of immersion	0.577	^49^
12	4	Normal clothing + immersion dry suit	Rest, head-out/first 3 min of immersion	0.6	^50^
13	4	Normal clothing + immersion dry suit	Rest, head-out/first 3 min of immersion	0.52	^50^
14	28	Swimming costume	Rest, head-out. First 2 min of immersion	0.5	^51^
15	10	Swimming costume	Flume swimming (completed)	2.2	^12^
16	18	Swimming costume	Flume swimming (completed)	2.43	^12^

HUE: helicopter underwater escape.

When drowning, it is unlikely that an individual will be ‘at rest’. It follows that the $\dot{V}$ O_2_ values most likely to represent a person attempting to stay afloat, get back to the surface of the water, or release a seat belt in a ditched vehicle and escape, will be those in Table 2 where activity coexists with cold immersion. In the situations where someone has to struggle to free themselves from debris or a harness and get back to the surface, an idea of the possible *maximum* $\dot{V}$ O_2_ can be obtained from that recorded during arm cranking (upper body activity). The average figures for this are 2.55 L.min^−1^ (34.2 mL . kg^−1^.min^−1^) for men and 1.81 L.min^−1^ (29.2 mL . kg^−1^.min^−1^) for women.^52^

Using rebreathing, Tipton et al.^23,24^ measured the breath by breath change of PAO_2_ and PACO_2_ in individuals wearing immersion suits undertaking a simulated helicopter underwater escape in water at 5 and 15°C. The average oxygen concentration at the end of the 1 min immersion was 7.69 kPa (57.7 mmHg) across conditions, with one individual falling to 4.9 kPa (36.8 mmHg) and remaining conscious. The corresponding average figure for carbon dioxide was 6.56 kPa (49.2 mmHg). Using the rates of reduction of oxygen concentration presented in this study, the average time to a PAO_2_ of 5.07 kPa (38 mmHg) was approximately 75 s. This duration would be extended if a larger breath were taken initially. However, as noted, in a drowning situation, air in the lung will be replaced by water with a break of breath-holding, reducing oxygen availability and therefore time to unconsciousness.

**Figure 2.**
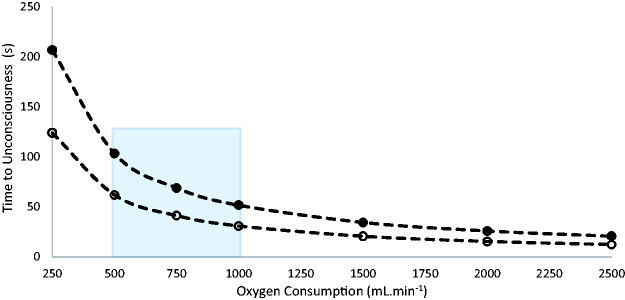
Relationship between oxygen consumption (aerobic physical activity) and time to unconsciousness during drowning for a 70 kg human at a lung capacity of 5.5 L in one atmosphere. Maximum volume of oxygen available = 1724 mL, loss of consciousness assumed when 50% of available oxygen has been used (see text). Solid circles = all air in lung available. Open circles = no air in lung available. Blue region represents likely intensities of exercise (Table 2).

Figure 2 shows the average relationship between $\dot{V}$ O_2_ and time to unconsciousness, assuming that this occurs when 50% of the available oxygen reserve of the body has been consumed (see discussion above). There are estimations and assumptions inherent in this figure, but it acts as a rough guide to the time of useful consciousness during drowning. Two lines are presented, one with and one without the oxygen available in the lung to represent those who manage to breath-hold for at least some of the period of submersion and those who cannot.

An alternative way of attempting to determine time to loss of consciousness is to examine studies of drowning. As discussed above, Carballo-Fazanes et al.,^17^ in their study of 24 drownings, noted that all persons drowned within 2 min. In Fainer et al.’s^53^ study of resuscitation from fresh water drowning in 160 mongrel dogs (weight 4.5 to 7.7 kg), a constant feature was the ‘cessation of struggling’ after an average 71 s of submersion (range for 95% of cases was 45 to 106 s). It cannot be confirmed whether the cessation of struggling and loss of consciousness coincide, or whether the results can be applied to cases of human drowning. However, the similarity between the estimated time to loss of consciousness in human studies (75 s^23^) and that reported by Fainer et al.^53^ (71 s) is noteworthy, if speculative. Other limited evidence comes from times to specific saturations of peripheral oxygen (S_p_O_2_) in patients undergoing general anaesthesia.^54^ The relevance of this literature to drowning is limited by the health, pre-oxygenation and low metabolic rate of anaesthetised patients; however, the most relevant cases (no pre-oxygenation, normal weight) suggest a time of about 60 s to reach a SpO_2_ of around 89%, albeit in anaesthetised patients with consequentially low $\dot{V}$ O_2._^54^ It is likely that a fully conscious, struggling individual would become more desaturated in 1 min.

It is concluded that the time to unconsciousness in a drowning individual will be dependent on the available oxygen stores within the body and the $\dot{V}$ O_2_ on submersion. Both of these values are variable, but the $\dot{V}$ O_2_ on submersion is likely to be significantly higher than the rates seen at rest, with the time to unconsciousness being consequently reduced, on average, to around 75 s (Figure 2).

### Time to cardio-respiratory arrest

**Figure 3.**
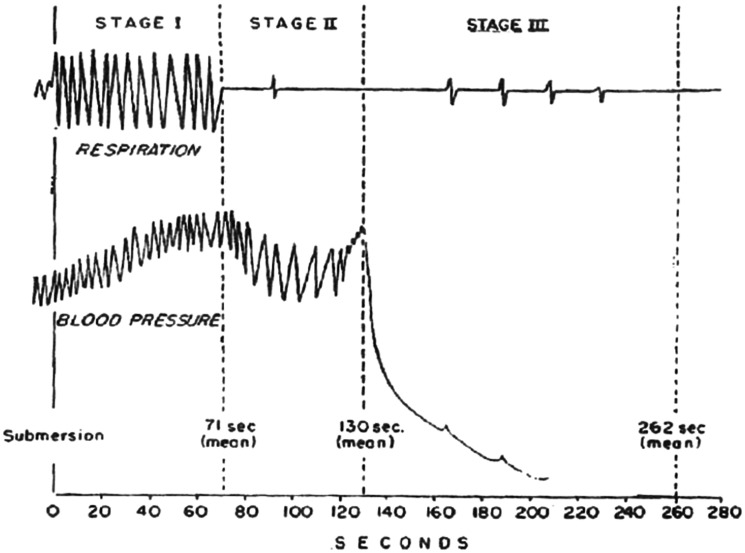
The stages of fresh water drowning in the dog. See text for details. From Fainer et al.^53^ with permission.

In the study of Fainer et al.,^53^ three stages of fresh water drowning were identified (Figure 3): (1) From submersion to cessation of struggling (see previous section). (2) From cessation of struggling to a precipitous fall in blood pressure – this occurred on average 130 s after submersion (range for 95% of cases was 78 to 160 s). Apnoea or irregular respirations occurred in this stage, and the blood pressure was characterised by slow ‘vagal-like’ beats. (3) From the precipitous fall in blood pressure until blood pressure reached zero. On average, this occurred 262 s after submersion (range for 95% of cases was 134 to 360 s). Terminal gasps occurred during this stage and sometimes after blood pressure reached zero (Figure 3).

### Death – inability to revive

The consensus in the literature is that the sooner a submerged, drowning individual is removed from the water and oxygenation and basic life support commenced, the better the prognosis. Less than 5–10 min of submersion has been associated with a better outcome by several authors.^55–58^ Claesson et al.^57^ reported that, following cardiac arrest due to drowning, all survivors at one month were found within 20 min, and 75% within 10 min. Quan et al.^58^ (2014) reported that of those with good outcomes following a drowning incident, 88.2% were submerged less than 6 min, 7.4% between 6 to 10 min and 4.3% for longer than 11 min. The longest submersion survived was 27 min. Szpilman et al.^6^ concluded that the risk of death or severe neurological impairment after hospital discharge is ‘nearly 100%’ when the duration of submersion exceeds 25 min.

Cerebral activity and therefore metabolism and oxygen demand fall by 6 to 7% for each 1°C fall in deep body temperature,^59^ becoming minimal at a brain temperature below 22°C.^60^ An exception to the ‘inability to revive’ times presented above can occur when drowning takes place in very cold water (<6°C). In this scenario, the aspiration of water may, by cooling the lungs, heart and blood supply to the brain, produce selective brain cooling and thereby extend the hypoxic survival time to over 60 min.^10,61^ The survivors of prolonged submersion tend to be children or small adults who, following the period of respiratory cooling, lose heat more quickly from the surface of the body due to a relatively high surface area to mass ratio.

## Conclusion

In this review, we have attempted to provide the information necessary to estimate the duration, and pain and suffering associated with drowning in different scenarios. The aspiration of water, oxygen reserves, rate of oxygen consumption and time spent without oxygen determine these aspects of drowning. They have not been considered together in the academic literature, but are often areas of detailed argument in legal cases that find their way to court.

## Dedication

This review is dedicated to the memory of Oscar Montgomery – a very fine young man and lover of the sea.
